# Electronic Health Record Messaging Patterns of Health Care Professionals in Inpatient Medicine

**DOI:** 10.1001/jamanetworkopen.2023.49136

**Published:** 2023-12-26

**Authors:** William Small, Eduardo Iturrate, Jonathan Austrian, Nicholas Genes

**Affiliations:** 1 New York University Grossman School of Medicine, New York, New York

## Abstract

This cross-sectional study examines secure messaging patterns among health care professionals in the internal medicine inpatient setting.

## Introduction

Miscommunication and teamwork failures are leading causes of sentinel events^1^ and cost US hospitals an estimated $12 billion in 2010.^2^ Information technology facilitates communication among health care professionals (HCPs). The US Centers for Medicaid & Medicare Services states that HCPs should only text patient information using secure messaging applications.^3^ Messaging is reported to improve patient emergency department length of stay.^4^ However, messaging has unintended patient safety consequences, such as increased interruptions for HCPs.^5^ Implementors of messaging technologies must appreciate interprofessional communication patterns, potential misuse, and consequences for patient care.

Since 2017, our large urban academic medical center has provided secure messaging access via desktop computers and smartphones. Patients can be linked to conversations comprising 2 or more HCPs in Epic Secure Chat, facilitating care decisions. To better understand use patterns among HCPs and relative consequences for their daily work, this study characterizes a mature electronic health record–based secure messaging network used in the care of internal medicine inpatients.

## Methods

The New York University Grossman School of Medicine Institutional Review Board approved this cross-sectional study and waived informed consent. The STROBE reporting guideline was followed. We extracted Epic Secure Chat metadata, including user details and time stamps, associated with internal medicine service inpatients (excluding intensive care unit [ICU] transfers) at 4 hospitals from January 25, 2021, to January 25, 2022. Roles were categorized as medicine and nonmedicine attendings, house staff, advanced practice professionals (eg, nurse practitioners and physician assistants), nurses, technicians, and social workers, care managers, or allied health professionals. User-level analysis included only HCPs who participated in at least 100 messages during the study period. Demographic information was limited for age and sex and was unavailable for race and ethnicity. Data analysis was performed on October 30, 2023, using Python, version 3.9.16 (GCC).

## Results

**Figure 1.**
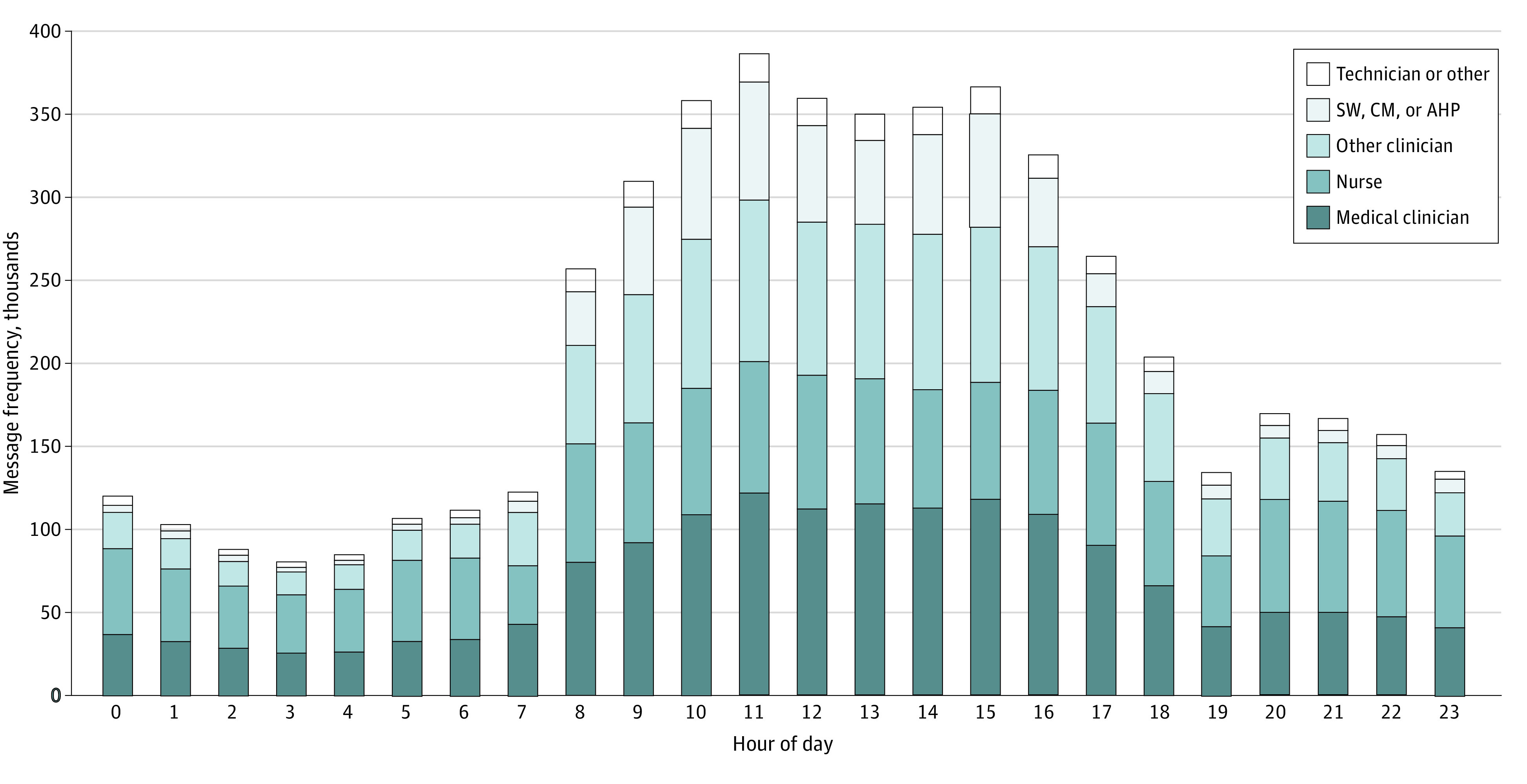
Hourly Message Frequencies, Stratified by Sender Role AHP indicates allied health professional; CM, care manager; SW, social worker.

This study included 14 329 participants. Over 12 months, 15.1 million messages were sent to 2.3 users each over 108 000 inpatient encounters (33.7 daily messages/encounter). There were 5.1 million messages sent about internal medicine inpatients to 2.5 users each during 22 900 encounters (35.9 daily messages/hospitalization). Approximately 69.1% of messages were sent between 8 am and 6 pm (Figure 1), and weekday volume was 36.3% higher. Most messages (75.9%) yielded responses within 5 minutes.

**Figure 2.**
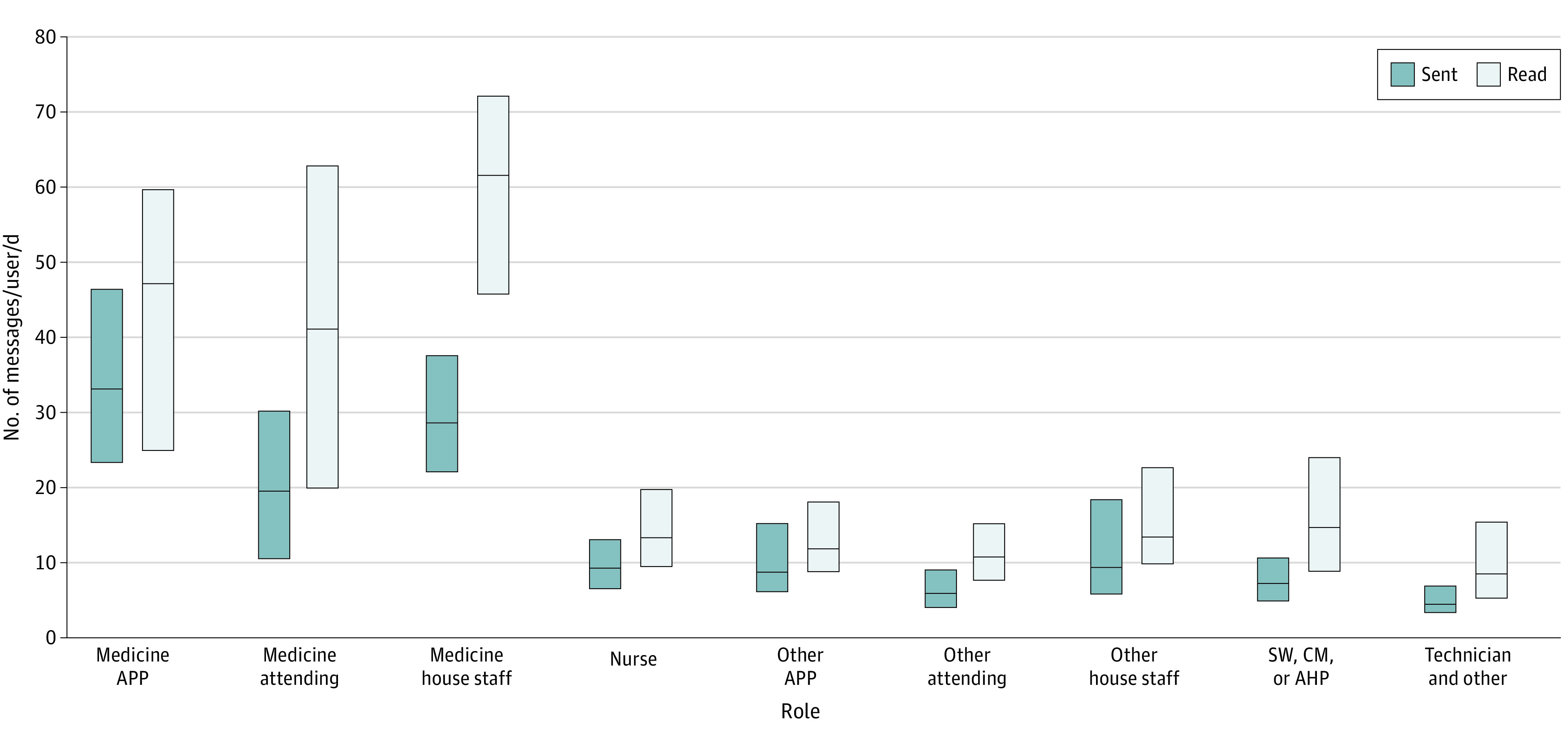
Variation in Daily Secure Messaging Usage Among Health Care Professionals, by Role Box plots illustrate the distribution of daily secure messages sent and read per user, grouped by unique user-role labels. Boxes represent the IQR with the median marked by a horizontal line. AHP indicates allied health professional; APP, advanced practice professional; CM, care manager; SW, social worker.

Nurses sent the largest proportion of messages (27.7%), followed by medicine house staff (13.5%) and social workers, care managers, and allied health professionals (12.6%). However, temporal variation existed, with medical clinicians sending the most daytime messages (Figure 1). Per user, medicine practitioners had the largest daily messaging burden (Figure 2). Whereas most medicine house staff (77.0%) received more than 45 daily messages, medicine attendings generally sent fewer messages but received up to 250 daily messages.

## Discussion

These findings suggest that messaging patterns differ by role, with medicine providers sending and receiving 2.8 and 3.1 times the average daily messaging volume as the other groups, respectively. Unlike the other groups, nursing did not exhibit diurnal messaging patterns and represented the largest contributor among roles despite lower per-user messaging. Future studies should evaluate consequences of messaging on individual and team performance, identify harmful patterns, and analyze message content to contextualize important events (eg, ICU transfers).

Institutional policies on storing messages limited our ability to analyze their content, which is critical to further interpreting inpatient communication patterns. Generalizability of these findings may be limited to academic medical centers with years of messaging experience.

Although secure messaging use has surged, concerns exist regarding its contributions to HCP interruptions^5^ and burnout analogous to in-basket messaging for outpatient HCPs.^6^ We cannot yet comment on adaptive messaging behaviors but emphasize that Epic Secure Chat should not be used for urgent and emergent issues; this should ease expectations of fast responses and promote HCP concentration. Characterizing messaging patterns in the inpatient setting represents a first step in facilitating benchmarking and, ultimately, strategic interventions to enhance the HCP digital experience, preserving the technology’s benefits while mitigating its disruptions.

## Notes

Supplement.
Data Sharing Statement
