# Supplementary material for: Electronic Health Record Messaging Patterns of Health Care Professionals in Inpatient Medicine
Source: JAMA Netw Open. 2023 Dec 26;6(12):e2349136. doi: 10.1001/jamanetworkopen.2023.49136 (PMC10751588; doi:10.1001/jamanetworkopen.2023.49136)
Supplement: Supplement. — Data Sharing Statement [file jamanetwopen-e2349136-s001.pdf]

Data Sharing Statement

Small. Electronic Health Record Messaging Patterns of Health Care Professionals in Inpatient Medicine. *JAMA Netw Open*. Published December 26, 2023.
doi:10.1001/jamanetworkopen.2023.49136

Data
Data available: No

Additional Information
Explanation for why data not available: Thank you for your interest in our research letter. We want to ensure transparency while prioritizing institutional data privacy and employee security. Regrettably, we cannot share private institutional data due to its sensitive nature. This data includes proprietary, confidential, or restricted information essential to our operations and security. We remain committed to scientific progress through responsible data sharing and are pleased to offer summary statistics and analysis code for the primary data upon request. We're open to discussing access to these elements for collaborative purposes. We appreciate the potential benefits of data sharing and are open to collaboration avenues that honor our strong commitment to data protection and confidentiality. For inquiries or more information, feel free to contact us.
